# TGF-b2 Induction Regulates Invasiveness of *Theileria*-Transformed Leukocytes and Disease Susceptibility

**DOI:** 10.1371/journal.ppat.1001197

**Published:** 2010-11-18

**Authors:** Marie Chaussepied, Natacha Janski, Martin Baumgartner, Regina Lizundia, Kirsty Jensen, William Weir, Brian R. Shiels, Jonathan B. Weitzman, Elizabeth J. Glass, Dirk Werling, Gordon Langsley

**Affiliations:** 1 Institut Cochin, Université Paris-Descartes, CNRS (UMR 8104), Paris, France; 2 Inserm U1016, Paris, France; 3 Université Paris-Diderot Paris 7, Paris, France; 4 CNRS UMR7216 Epigénétique et Destin Cellulaire, Paris, France; 5 University of Berne, Department of Clinical Research and Veterinary Public Health, Division Molecular Pathobiology, Bern, Switzerland; 6 Royal Veterinary College, Hawkshead Lane, North Mymms, Hatfield, Hertfordshire, United Kingdom; 7 Division of Genetics and Genomics, The Roslin Institute and Royal Dick School of Veterinary Studies, The University of Edinburgh, Roslin, Midlothian, United Kingdom; 8 Division of Veterinary Infection and Immunity, University of Glasgow, Faculty of Veterinary Medicine, Institute of Comparative Medicine, Bearsden Road, Glasgow, United Kingdom; Bernhard Nocht Institute for Tropical Medicine, Germany

## Abstract

*Theileria* parasites invade and transform bovine leukocytes causing either East Coast fever (*T. parva*), or tropical theileriosis (*T. annulata*). Susceptible animals usually die within weeks of infection, but indigenous infected cattle show markedly reduced pathology, suggesting that host genetic factors may cause disease susceptibility. Attenuated live vaccines are widely used to control tropical theileriosis and attenuation is associated with reduced invasiveness of infected macrophages *in vitro*. Disease pathogenesis is therefore linked to aggressive invasiveness, rather than uncontrolled proliferation of *Theileria*-infected leukocytes. We show that the invasive potential of *Theileria*-transformed leukocytes involves TGF-b signalling. Attenuated live vaccine lines express reduced TGF-b2 and their invasiveness can be rescued with exogenous TGF-b. Importantly, infected macrophages from disease susceptible Holstein-Friesian (HF) cows express more TGF-b2 and traverse Matrigel with great efficiency compared to those from disease-resistant Sahiwal cattle. Thus, TGF-b2 levels correlate with disease susceptibility. Using fluorescence and time-lapse video microscopy we show that *Theileria*-infected, disease-susceptible HF macrophages exhibit increased actin dynamics in their lamellipodia and podosomal adhesion structures and develop more membrane blebs. TGF-b2-associated invasiveness in HF macrophages has a transcription-independent element that relies on cytoskeleton remodelling via activation of Rho kinase (ROCK). We propose that a TGF-b autocrine loop confers an amoeboid-like motility on *Theileria*-infected leukocytes, which combines with MMP-dependent motility to drive invasiveness and virulence.

## Author Summary

*Theileria annulata* causes tropical theileriosis that is endemic in cattle in North Africa, the Middle East, India and China. *T. parva* causes East Coast fever that is prevalent in East and Southern Africa. In endemic countries indigenous cattle are more resistant to pathology, but produce little meat and milk and attempts to improve output by importing European and American breeds have failed due to a high susceptibility to these diseases that are often rapidly fatal. We examined *T. annulata*-transformed macrophages isolated from disease resistant Sahiwal compared to disease-susceptible Holstein-Friesian (HF) cattle, for their capacity to traverse synthetic extra-cellular matrix *in vitro*. The invasive capacity of all transformed macrophages was TGF-b dependent, but those of disease-susceptible HF animals invaded better *i.e.* they were more aggressive. The greater invasive capacity of HF transformed macrophages matched their increased production of TGF-b2, since levels of TGF-b1, and all three TGF-b receptors, were the same as in transformed macrophages isolated from disease-resistant Sahiwal animals. TGF-b2 production therefore likely renders *Theileria*-transformed leukocytes more pathogenic and consistently, in a live attenuated line used to vaccinate against tropical theileriosis transcripts of *TGF-b2* and those of a significant number of TGF-target genes drop and consequently, TGF-b-mediated invasiveness decreases.

## Introduction

Cellular transformation is a complex, multi-step process and leukocyte transformation by *Theileria* is no exception, as parasite infection activates several different leukocyte-signalling pathways, the combination of which leads to full host cell transformation [1]. However, *Theileria*-induced leukocyte transformation is unusual in that it is rapid and appears to be entirely reversible with the host cell losing its transformed phenotype upon drug-induced parasite death [2]. Just like most cancer cells however, *Theileria*-induced pathogenesis (virulence) is associated with the invasive capacity of transformed leukocytes, which is lost upon attenuation of vaccine lines [3]. Attenuation of virulence has been ascribed to decreased matrix-metallo-proteinase-9 (MMP9) production and loss of AP-1 transcriptional activity [4]. Consistently, functional inactivation of AP-1 resulted in reduced tumour formation, when infected and transformed B cells were injected into Rag2gC mice [5].

Host leukocyte tropism differs with *T. parva* infecting all subpopulations of lymphocytes whereas *T. annulata* infects monocytes/macrophages, dendritic cells and B lymphocytes [1]. Despite this, the diseases they cause (called tropical theileriosis with *T. annulata* infection and East Coast fever with *T. parva* infection) are both severe, as susceptible animals usually die within three weeks of infection. The geographical distribution of their respective tick vector species determines areas where disease is widespread. Tropical theileriosis affects over 250 million animals and extends over the Mediterranean basin, the Middle East, India and the Far East, whereas East Coast fever is prevalent in eastern, central and southern Africa. It is noteworthy that in endemic areas indigenous breeds of cattle are more resistant to disease. For example, when *Bos indicus* Sahiwals are experimentally infected with *T. annulata* they exhibit fewer clinical symptoms and recover from a parasite dose that is fatal in the European Holstein-Friesian (HF) *B. taurus* breed [6]–[7]. *Theileria*-infected leukocytes are capable of producing IL-1 and IL-6 [8], as well as GM-CSF [9] and TNF [10]. Nonetheless, no differences in the level of expression of the pro-inflammatory cytokines TNF, IL-1b, or IL-6 were detected between disease-resistant Sahiwal- versus HF-infected macrophages [11]. Some additional inherent genetic trait of Sahiwal animals must therefore underlie their disease-resistance. Although transcriptome analysis of 3–5 times passaged Sahiwal and HF macrophages following infection with *T. annulata* revealed significant breed differences in both the resting and infected gene expression profiles, no clear candidate genetic trait was revealed [12].

Transforming growth factor beta (TGF-b) is a family of cytokines and both TGF-b1 and TGF-b2 can bind with high affinity to the TGF-b type II receptor (TGF-RII) leading to the recruitment of TGF-RI. The constitutive kinase activity of TGF-RII phosphorylates and activates TGF-RI, which in turns recruits and activates Smad2 and Smad3, which bind Smad4, and the whole complex translocates to the nucleus and induces the transcription of target genes [13]. The TGF-b signalling pathway can be negatively regulated [14] and an increasing number of non-Smad-mediated TGF-b signalling pathways have been described [15]. TGF-b can also regulate cytoskeleton dynamics via transcription-dependent and transcription-independent processes [16]. It is likely that all these different pathways contribute in different ways to the pleiotropic effects of TGF-b (see http://www.cell.com/enhanced/taylor). TGF-b can exert opposite effects on cell growth: in most non-transformed cells TGF-b is usually growth inhibitory, but it can increase motility of certain mesenchymal cells and monocytes, but however, at some point in the transformation process TGF-b becomes pro-metastatic [17]–[18], for example in ovarian cancer [19]. We show here that TGF-b plays a role in infected host leukocyte invasiveness. Importantly, the high level of TGF-b2 production by *Theileria*-infected HF-transformed macrophages renders them more invasive than those of disease-resistant Sahiwal animals. In addition, vaccination against tropical theileriosis uses live attenuated *T. annulata*-infected macrophages and attenuation leads to the loss of both *TGF-b2* transcription and alteration in the expression of a set of TGF-b-target genes, and a drop in TGF-b-mediated invasion. Thus, *Theileria*-dependent TGF-b2 induction is a virulence trait that underscores susceptibility to tropical theileriosis.

## Results

### Induction of *TGF-b2* by *T. annulata* is greater in infected macrophages isolated from disease-susceptible compared to disease-resistant bovine hosts

**Figure 1:**
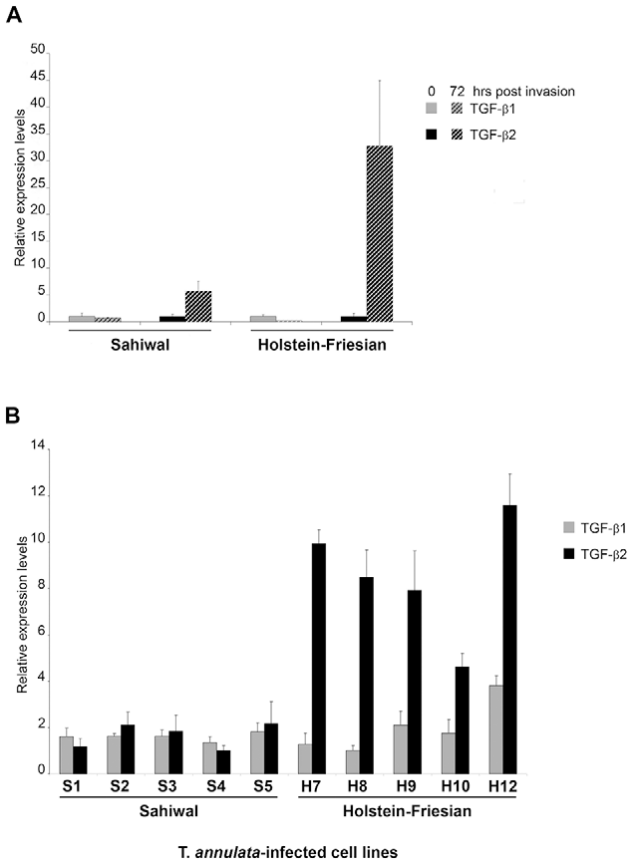
Induction of *TGF-b2* by *T. annulata* is greater in infected macrophages isolated from disease-susceptible compared to disease-resistant bovine hosts. **A**, Macrophages isolated from Sahiwal and HF cows were infected *in vitro* with sporozoites of the Hissar strain of *Theileria annulata*. RNA was isolated immediately prior to infection (0h) and 72h post-infection and the relative amounts of *TGF-b1* and *TGF-b2* transcript measured by real-time RT-PCR. **B**, Total RNA was extracted from *T. annulata*-infected cell lines obtained from infection of Sahiwals (S1–5) and HF (H7–12) animals. *TGF-b1* and *TGF-b2* relative mRNA levels were determined by real-time RT-PCR. The relative value obtained for the cell line showing the lowest expression of each cytokine (H8 for TGF-b1 and S4 for TGF-b2) was used for comparison and arbitrary set at 1. Error bars represent standard deviations for triplicate samples. No significant difference between Sahiwal and Holstein-Friesian cells was found for *TGF-b1* (p = 0.710), in contrast to *TGF-b2* that was 7-fold upregulated (p<0.001) in all 5 H cells lines.

As *Theileria*-transformed leukocytes are known to secrete a number of different cytokines we examined whether infection by *T. annulata* sporozites of the same parasite strain (Hissar) could induce *TGF-b* in macrophages 72h post-invasion, as described [12]. Prior to infection both Sahiwal and HF macrophages produced low levels of *TGF*-*b* transcript with slightly higher amounts of *TGF-b1* (Fig. 1A). Interestingly, *Theileria* infection induces preferentially *TGF-b2* in both Sahiwal and HF macrophages, and importantly, the induction after 72h is much greater in disease-susceptible HF cells.

We next examined the levels of *TGF-b* transcripts in a series of *T. annulata*-transformed cell lines derived from Sahiwal and HF animals. Again, *TGF-b1* and *TGF-b2* mRNA could be detected in all 10 transformed cell lines (Fig. 1B). Similar to freshly invaded cells the relative mRNA levels of *TGF-b1* did not differ significantly across the *T. annulata* infected cell lines and there was no evidence of a breed-specific difference in *TGF-b1* expression (Fig. 1B grey bars, p = 0.710). In contrast, the relative *TGF-b2* mRNA levels exhibit statistically significant differences (Fig. 1B black bars, p<0.001), with *TGF-b2* mRNA levels being higher in HF cell-lines. Additional qRT-PCR experiments revealed that the levels of expression of *TGF-RI*, *TGF-RII* and *TGF-RIII* were equivalent in HF versus Sahiwal *T. annulata*-infected cell lines (data not shown). Thus, disease susceptibility correlates to the level of *TGF-b2* transcripts that are expressed 7-fold (p<0.001) more by *T. annulata*-transformed macrophages of HF origin.

### The invasive capacity of *Theileria*-transformed HF macrophages is greater than Sahiwal-infected macrophages and is TGF-b-dependent

**Figure 2:**
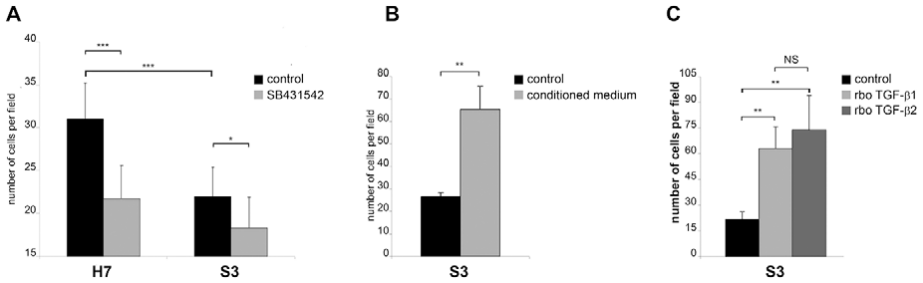
The invasive capacity of *Theileria*-transformed HF macrophages is greater than Sahiwal-infected macrophages and is TGF-b-dependent. **A**, The invasive capacity of H7 and S3 cells was determined by Matrigel chamber assays. Seven independent experiments were performed and each sample counted three times. The y-axis is the number of migrated cells for 10 independent fields. **B**, Conditioned medium from H7 cultured cells stimulates the invasive capability of S3 cells (*** p<0.0005). Invasion assay was performed on untreated S3 cells and S3 cells that had been incubated for 24h prior to the assay with culture supernatants from H7 cells grown overnight in low-serum (0.5% FCS) containing media. **C**, Addition of recombinant TGF-b1 and TGF-b2 leads to an increase in the invasive index of *Theileria*-infected S3 macrophages (** p<0.005).

In *T. parva*-transformed B cells a TGF-b-mediated signalling pathway is active and invasion is partially TGF-b-dependent (Fig.S1). We therefore compared the invasive capacity of *T. annulata*-transformed HF versus Sahiwal macrophages (Figure 2). We found that disease-susceptible HF macrophages displayed 30% greater capacity (p<0.005) to traverse Matrigel than infected Sahiwal macrophages and that traversal is again TGF-b-dependent (Fig. 2A). The invasive capacity of the H7 cell line was reduced to levels equivalent to S3 cells upon treatment with the TGF-R inhibitor (Fig. 2A) and conversely, when S3 cells were stimulated with conditioned medium from H7 cultures, S3 cells displayed increased invasiveness (Fig. 2B). Moreover, the reduced invasive capacity of disease-resistant S3 macrophages could be restored to above virulent levels by addition of either TGF-b1, or TGF-b2 (Fig. 2C), demonstrating that the TGF-b signalling pathways are intact in these cells. *Theileria* infection therefore preferentially induces up-regulation of TGF-b2 and increased invasiveness of transformed leukocytes.

### *In vitro* attenuation is associated with lower TGF-b2 expression and altered transcripts levels of TGF-b-regulated genes

**Figure 3:**
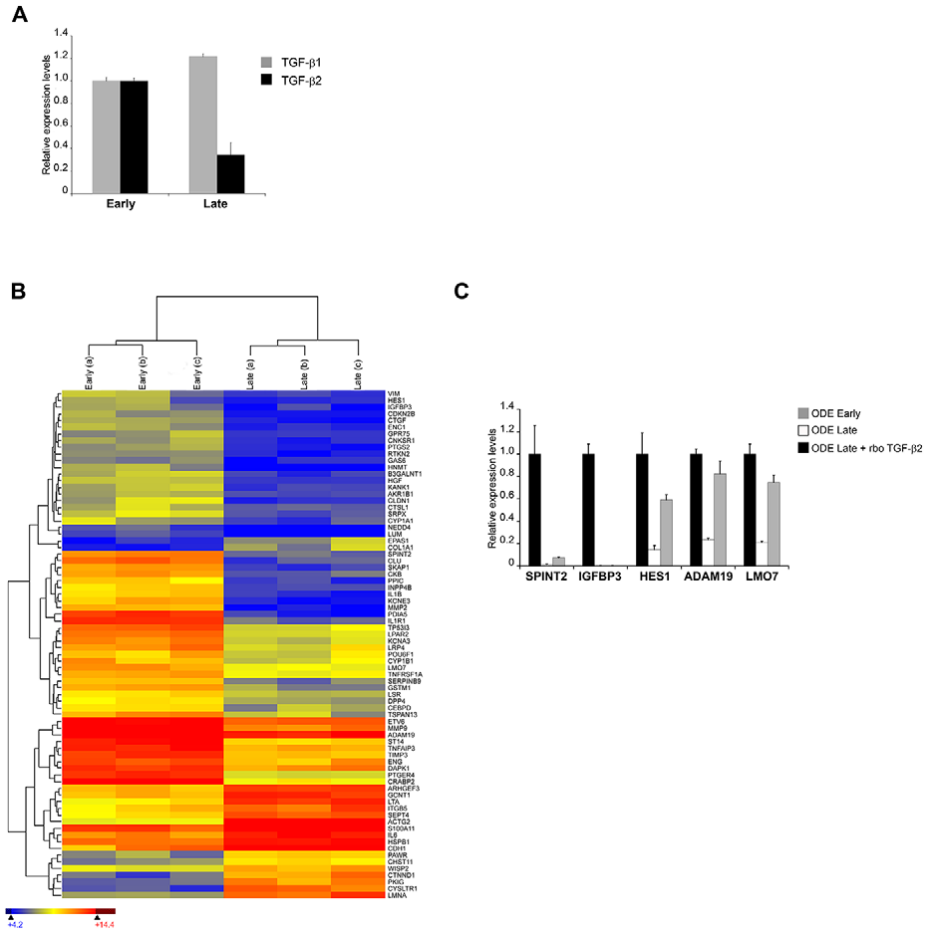
*In vitro* attenuation is associated with lower TGF-b2 expression and altered transcripts levels of TGF-b-regulated genes. **A**, Total RNA was extracted from early and late passage ODE cells. *TGF-b1* and *TGF-b2* relative mRNA levels were determined by real-time RT-PCR. **B**, Total RNA from early and late passage ODE cells (3 replicates each) was hybridised to a microarray representing known bovine genes, including 1,158 targets of TGF-b. 76 of these genes were identified as differentially expressed using the criteria of a false discovery rate <5% (Rank Product analysis) and a fold change >2. Hierarchical clustering was performed on this gene set and the results are depicted as a heat-map. The gene symbol is shown on the right of the heat-map, where colour is used to represent gene expression level: blue (low), yellow (intermediate) and red (high). **C**, Total RNA was extracted from early and late passage ODE cells, as well as late passage ODE cells treated for 24h with rboTGF-b2 (5ng/ml). Relative mRNA levels for the indicated genes were determined by real-time RT-PCR and normalized to HPRT1 relative levels.

*T. annulata*-infected cell lines can be attenuated for virulence by multiple *in vitro* passages to generate live vaccines that are used to protect against tropical theileriosis [3]. The molecular basis of attenuation is not known, but our above observations on preferential TGF-b2 induction and augmented host cell invasiveness suggest that attenuation might be lead to reduced *TGF-b2* transcription and TGF-b2-mediated invasion. To directly test this prediction we examined the Ode vaccine line derived from an infected HF cow in India [20] and estimated the level of *TGF*-*b2* transcripts and TGF-b-target gene transcription in virulent (early passage) and attenuated (late passage) infected macrophages (Figure 3). As predicted, attenuation leads to a significant decrease in the amount of *TGF-b2* message and surprisingly, a slight increase in *TGF-b1* transcripts (Fig. 3A). This strongly suggests that upon attenuation the parasite's ability to induce host cell *TGF-b2* has been impaired. Reduced levels of TGF-*b*2 should lead to an alteration in the transcription profiles of known *TGF-b*-target genes and to see if this is indeed the case, we performed microarray analyses and hierarchical clustering of transcript levels. The microarray representing 26,751 bovine genes included 1,158 targets of TGF-b (http://www.netpath.org/) and 76 of these genes were identified as differentially expressed upon attenuation. The heat-map, where low gene expression level is depicted as blue, intermediate as yellow and high expression as red, is present in Fig. 3B. Among the down-regulated TGF-target genes, five were chosen at random and their expression verified by qRT-PCR using mRNA from early and late passage Ode (Fig. 3C). In each case their transcription was reduced upon attenuation and could be restored by adding exogenous recombinant TGF-b2. Consistently, their expression was high in disease-susceptible infected HF macrophages that produce more TGF-b2 (see Fig. 1) and low in Sahiwal macrophages, but could be augmented by exogenous TGF-b2 stimulation (Fig.S2). Attenuation of virulence therefore, leads to ablation of TGF-b2 signalling and an alteration in the profile of expression of a set of TGF-target genes.

### Invasiveness is reduced upon *in vitro* attenuation of host cell virulence

**Figure 4:**
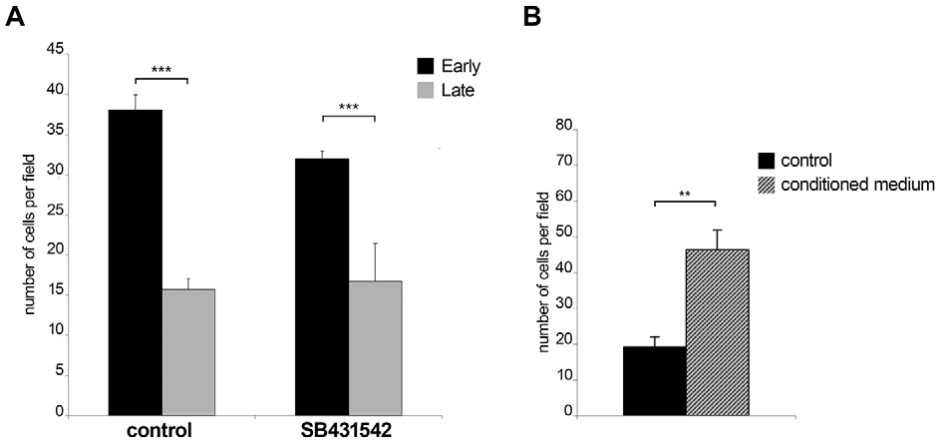
Invasiveness is reduced upon *in vitro* attenuation of host cell virulence. **A**, Early passage Ode cells display higher invasive capability compared to late passage Ode (***p<0.005). The invasive capacity of early passage Ode is reduced (***p<0.005) upon TGF-R blockade by SB431542 **B**, Culture supernatant from early passage ODE cells stimulates the invasive capability of late passage ODE cells (**p<0.005). Invasion assay was performed on untreated late passage ODE cells and late passage cells that had been incubated for 24h prior to the assay with culture supernatants from early passage ODE cells grown overnight in low-serum (0.5% FCS) containing media.

The observation that early passage Ode cells express higher levels of *TGF-b2* message and have altered expression of 76 TGF-b-target genes led us to compare early with late passage Ode and examine the contribution of TGF-b to their invasive capacity (Figure 4). As previously described [4], attenuation of Ode leads to a significant drop in invasive capacity (***p<0.005) and receptor blockade by SB431542 gives an estimate (***p<0.005) of the contribution of TGF-b to early passage Ode virulence (Fig. 4A). The potential contribution of TGF-b to virulence has been ablated by attenuation, since the invasive capacity late passage Ode is insensitive to receptor blockade (Fig. 4A, right). When conditioned medium from early passage Ode is given to late passage Ode there is a marked (**p<0.05) regain in invasion (Fig. 4B). Virulent Ode therefore, secretes factors into the medium that contribute to invasiveness, one of which is clearly TGF-b2, and this capacity is lost upon attenuation. The partial inhibition of invasion by early passage Ode by SB431542 might also suggest that although virulent following 65/70 *in vitro* passages some attenuation of TGF-b2 induction might be occurring.

### TGF-b promotes podosomal adhesion structures and ROCK-mediated cortical actin-rich membrane blebs

**Figure 5:**
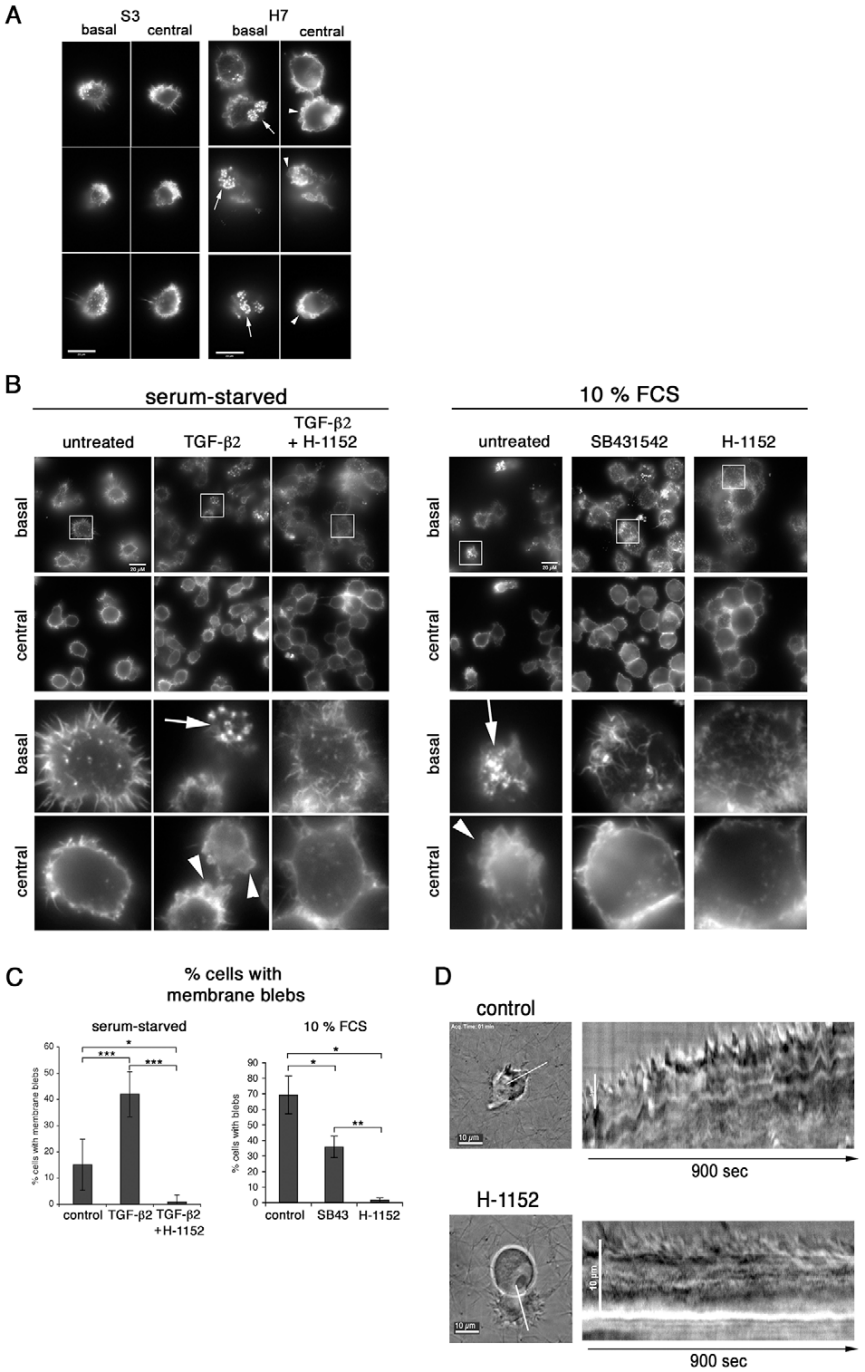
TGF-b promotes podosomal adhesion structures and ROCK-mediated cortical actin-rich membrane blebs. **A**, S3 and H7 cells were seeded onto a gelatin/fibronectin matrix. After 24h in culture, the actin cytoskeleton was visualized by IF microscopy. Basal and central focal planes are shown and actin structures were visualized by phalloidin staining at basal adhesion sites and in the lateral cortex, respectively. **B**, Left panel: H7 cells were serum starved in 0.5% FCS for 24h and then seeded onto a gelatin/fibronectin matrix in starvation medium and in the absence or presence of TGF-b2 (10ng/ml) and H-1152 (10uM), as indicated. Right panel: H7 cells were grown in complete growth medium and then seeded onto a gelatin/fibronectin matrix in complete growth medium (10% FCS) and in the absence or presence of SB431542 and H-1152, as indicated. Analyses was performed as described in A. Magnifications in lower half of the panels are four-fold of areas highlighted with frames. **C**, Statistical analysis of experiment shown in panel B. Percentages of cells with membrane blebs within total cell populations was determined. Every cell in at least four independent areas per condition was analysed. 0.5% FCS: control: n = 121 (15.11%−/+9.7), TGF-b: n = 235 (42.00%−/+8.5), TGF-b+H-1152 N = 143 (1%); 10% FCS: control: n = 60 (69.27%−/+12.12), SB431542 = SB43: n = 167 (35.84%−/+6.94), H-1152: n = 81 (1.66%−/+1.47). Arrows indicate podosomal adhesion structures, arrowheads membrane blebs. **D**, Infected cells were embedded in fibrillar collagen and live-cell video microscopy was performed 24h after embedding. Where indicated, 10uM Rho-kinase inhibitor H-1152 was added 1h before acquisition of movies. Sill images of movies and kymographs along the white line indicated are shown.

We next studied whether the TGF-b-mediated invasion programme might have a consequence on cellular adhesion and invasion structures such as lamellipodia, podosomes and membrane blebs. We first investigated by time-lapse video microscopy lamellipodia morphology of *T. annulata*-infected macrophages cultured without (control), or with SB431542 (Fig.S3 and Movies S1 and S2) and found that the size of lamellipodia (shown boxed) decreased upon SB431542 treatment (Fig.S3A). Visualisation of the actin cytoskeleton with Texas red-labelled phalloidin showed that decreased lamellipodia size correlated with reduced actin dynamics (Fig.S3B and C) and suggested that TGF-controls cortical actin dynamics in infected macrophages. We next compared the basal and central cortical actin cytoskeleton of S3 and H7 cells cultured on a gelatin/fibronectin matrix, which facilitates adhesion of these cells. In S3 cells, we observed only small podosomal adhesion structures that were rarely clustered and no actin-rich membrane blebs (Fig. 5A). In contrast, in H7 cells podosomal adhesion structures were markedly enlarged and clustered and the majority of cells displayed actin-rich membrane blebs. Membrane blebbing was confirmed by live-cell imaging (Fig.S4A), which highlights the dynamics of bleb formation. Individual blebs expand within one to three seconds and persist for approximately 30–120 seconds, which is a time frame typically observed in bleb formation and retraction [21]. Reducing the serum concentration from 10% to 0.5% (starvation) resulted in a significant decrease in the number of membrane blebs (Fig. 5B and C). Membrane blebbing in starved cells was rescued by the exogenous addition of TGF-b2. The TGF-b-induced membrane blebs were blocked by the Rho-kinase (ROCK) inhibitor H-1152 and in the presence of serum the formation of actin-rich membrane blebs was significantly reduced after treatment with the TGF-R inhibitor, but completely blocked in the presence of H-1152 (Fig. 5C). Thus, one consequence of increased TGF-b2 production is increased cortical actin dynamics, which likely gives rise to enhanced podosomal adhesion structures (invadosomes) and membrane blebs in macrophages derived from disease susceptible HF cattle.

We next investigated the functional significance of membrane blebs for cell motility in 3-D matrices. In the low rigidity fibrillar collagen or high-density Matrigel matrices, H7 macrophages acquired an amoeboid pattern of motility with characteristic polarized formation of membrane blebs (Fig. 5D and Fig.S4B and C). Membrane bleb formation required ROCK activity and inhibition of ROCK prevents local contractibility, polarized bleb formation and forward movement of the cell. Taken together, these data show that exposure of H7 macrophages to TGF-b2, results in ROCK-dependent membrane blebbing that drives motility in 3-D matrices.

## Discussion

We have shown here that *Theileria*-induced leukocyte transformation results in the constitutive induction of a TGF-b autocrine loop that augments the invasive potential of infected leukocytes. We could find no evidence for a contribution of TGF-b signalling to host cell survival, or proliferation (data not shown), implying that leukocyte infection by *Theileria* essentially confers on TGF-b a pro-metastatic role. Smads and p53 are known to associate and collaborate in the induction of a subset of TGF-b target genes [22]. Recently, p53 has been described as being sequestered in the cytosol of *Theileria*-transformed leukocytes, as part of a parasite-induced survival mechanism [23]. Although not addressed by Haller *et al* it is possible that cytosol located p53 might ablate nuclear translocation of Smads, thus counteracting the anti-proliferative affect of TGF-b in *Theileria*-transformed leukocytes?

Importantly, comparison of disease-susceptible HF transformed macrophages to disease-resistant Sahiwal ones, showed that the degree to which *Theileria* (the same parasite strain) induces TGF-b2 influences the invasive potential of the infected and transformed host cell. The likelihood of developing a life-threatening cancer-like disease therefore appears to be due in part to the inherent genetic propensity of HF macrophages to produce high levels of TGF-b2 upon infection that might render the transformed macrophages more invasive. *T. annulata*-transformation of HF macrophages leads to the induction of higher amounts of *TGF-b2* the levels of induced *TGF-b1* mRNA being the same as in Sahiwal macrophages. As *TGF-RI* and *-RII* and *-RIII* [24] are expressed to the same extent in the two types of macrophages (data not shown) it suggests that only the amount of *TGF-b2* is crucial. The predisposition of *Theileria* transformation to induce *TGF-b2* over *TGF-b1* in HF versus Sahiwal macrophages implies that there could be disease-associated sensitivity to infection linked to TGF-b2 over-production. Species-specific promoter differences, or some other unknown breed difference may explain the greater propensity of *Theileria* to induce *TGF-b2* over *TGF-b1* transcripts in HF compared to Sahiwal cattle. However, promoter sequence differences seem unlikely to underlie *Theileria*'s ability to induce *TGF-b2* over *TGF-b1* transcripts, or explain the drop in *TGF-b2* levels upon attenuation of the Ode vaccine line as here, both virulent and attenuated infected macrophages are of HF origin [20]. Loss of virulence of the Ode vaccine line upon long-term *in vitro* passage is clearly associated with a decrease in *TGF-b2* transcripts and it would appear that the parasite's ability to specifically activate host cell transcription of *TGF-b2* is impaired and one possibility is that attenuation is associated with altered epigenetic regulation of *TGF-b2* promoter activity. Microarray analyses indicate that upon attenuation of virulence, not only do *TGF-b2* levels drop, but also 76 TGF-b-target genes display altered transcription. It would appear then that preferential TGF-b2 induction following *Theileria* infection initiates a host cell genetic programme that contributes to more aggressive invasiveness of transformed HF macrophages. We believe the same TGF-b2-initiated genetic programme also contributes to the invasiveness, albeit reduced, of disease-resistant Sahiwal macrophages, as *Theileria* infection also preferentially induces TGF-b2, just to a lesser extent.

We have used fluorescence and time-lapse video microscopy to examine the morphology of *Theileria*-infected Sahiwal and HF infected macrophages and the effect of TGF-b and Rho kinase (ROCK) on actin dynamics and lamellipodia formation. *Theileria* infected, disease-susceptible HF macrophages show increased actin dynamics in their lamellipodia and podosomal adhesion structures and a remarkable propensity to develop membrane blebs. Figure 5D shows the dynamic behaviour of infected cells embedded in 3-D matrices (see also Fig.S3 and movies S3 and S4), where either fibrillar collagen, or matrigel was used giving two 3-D matrices of low (collagen) and high (matrigel) rigidity. Membrane blebbing of motile H7 cells occurs in both matrices in a polarized fashion at the leading edge, clearly suggesting that membrane blebbing is required for infected cell motility in 3-D. Movie S3 shows bleb-driven membrane protrusions, which results in forward movement of the cell (see also kymographs of movie S3, Fig. 5D). Moreover, ROCK activity is required, because inhibition of ROCK with H-1152 impairs polarized bleb formation and forward movement in fibrillar collagen and matrigel. Spatio-temporal control of Rho-ROCK activity is also required for cell polarization and lamellipodia formation in 2-D [25]. Since TGF-b acts upstream of Rho-ROCK in infected cells, we would therefore predict that spatio-temporal activation of Rho-ROCK – controlled by TGF-b signalling – is required for lamellipodia formation as well. Combined, increased bleb and lamellipodia formation could give rise to more invadosomes on infected virulent macrophages in a similar manner to TGF-b-mediated increased adhesion of immortalised hepatocyte cell lines [26].

We showed that augmented invasiveness by TGF-b2 in disease-susceptible HF macrophages has a transcription-independent element that relies on cytoskeleton remodelling via activation of Rho and its downstream target ROCK [27]–[28]. Given the important role played by ROCK in increased blebbing of infected host cells and the recent description that Rho/ROCK signals amoeboid-like motility [29], it is tempting to speculate that the TGF-b autocrine loop confers on *Theileria*-infected leukocytes an amoeboid-like motility that contributes to invasiveness and makes them more virulent. However, since prolonged TGF-b stimulation can result in decreased Rho activity due to the action of p190RhoGap [30], *Theileria* infected cells must have developed a mechanism to balance TGF-b-induced RhoGAP activities. It is possible that the parasite might also regulate the expression, activity or localization of Rho-family GEFs, as the Rho activator GEF-H1/Lfc has been shown to be a TGF-b1 target gene [31] and an analogous mechanism involving TGF-b2 might be exploited by *Theileria* parasites? Alternatively, the parasite could function by excluding negative regulators of Rho from specific subcellular compartments, analogous to the exclusion of the RhoGAP Myo9b from lamellipodia of macrophages [25].

Whatever the underlying mechanism of inducing TGF-b2 levels in *Theileria*-infected leukocytes, its induction and the genetic programme it initiates is clearly correlated with the invasive phenotype of transformed macrophages of disease-sensitive hosts. This implies that overall invasiveness of *Theileria*-transformed leukocytes is made up of amoeboid (TGF-b- & ROCK-dependent) and mixed amoeboid/proteolytic (MMP-dependent [4]) motility; reviewed in [32]. These *Theileria*-based observations also suggest that in some cases the propensity of human leukaemia patients to develop life-threatening cancer could be due to the inherent genetic predisposition of their tumours to produce high levels of TGF-b2, rather than TGF-b1, and the genetic programme this initiates on promoting an additional amoeboid-like invasive phenotype of their tumours.

## Materials and Methods

### *Theileria*-infected cell lines

TpMD409.B2 is a *T. parva Muguga*-infected B-cell clone (B2) and its B-cell characteristics have been previously confirmed [33]. The cell lines S1–S5 and H7–H10 have been described previously [6]. *In vitro* infection of uninfected S and H cells by *Theileria* sporozoites was done as described [12]. The characterisation of the Ode vaccine line has been reported [20] and in this study virulent/early Ode corresponds to passage 65–70 and attenuated to passage 318–322. It is possible that passages 65–70 have already become slightly attenuated. All cultures were maintained in RPMI-1640 medium supplemented with 10% foetal calf serum (FCS) and 50 uM b-mercaptoethanol. Cell cultures were passaged 24h before harvesting to maintain the cells in the exponential growth phase.

### Inhibitors and reagents

The TGF-bRI/ALK5 inhibitor SB431542 (Sigma #S4317) was added at 10uM for 18h. The Rho kinase (ROCK) inhibitor H-1152 (Alexis Biochemicals, #ALX-270-423) was added at 10uM. Recombinant bovine TGF-b1 and TGF-b2 (rboTGF-b1 and rboTGF-b2; both NIBSC, Potters Bar. UK) were added in the culture media at 10ng/ml and incubated for different times (15 min or 30 min).

### Transfections, expression plasmids and reporter constructs

All transfections were carried out by electroporation as previously described [34]. The CAGA-luc (the Smad 3/4 binding element-luciferase construct) was transiently transfected into B2 cells with the inhibitory Smad7 plasmid (Flag-Smad7-pcdef3), or an empty vector (pcdef3). The major late minimal promoter (MLP - a minimal promoter consisting of the TATA box and the initiator sequence of the adenovirus major late promoter) was cloned into pGL3 (Promega) to generate the MLP-luc plasmid that was used as minimal promoter negative control [35]–[36]. Efficiencies of transfections were normalized to renilla activity by co-transfection of a pRL-TK renilla reporter plasmid (Promega #E6241). Luciferase assay was performed 24h after transfection using the Dual-Luciferase Reporter Assay System (Promega, #E1980) in a microplate luminometer (Centro LB 960, Berthold). Relative luminescence was represented as the ratio firefly/renilla luminescence.

### Western-Blot analysis

Nuclear extracts of from *T. parva*-infected (B2) cells were prepared as described [37]. 20ug of proteins were separated in a denaturing 8% SDS-PAGE gel and electro-transferred onto a nitrocellulose membrane (Scheicher and Schuell). Antibodies used in immuno-blotting were as follows: anti-phospho-Smad2 (Cell Signaling #3101), anti-Smad2 (BioVision, #3462-100), anti-PARP (Clone C2-10, Pharmingen #556362).

### Total RNA extraction

Total RNA was isolated from each of the *T. annulata* infected cell lines using the RNeasy mini kit (Qiagen) according to the manufacturer's instructions. The quality and quantity of the resulting RNA was determined using a Nanodrop spectrophotometer and gel electrophoresis and for microarray screens on an Agilent 2100 Bioanalyser (Agilent Technologies). mRNA was reverse transcribed to first-strand cDNA and the relative levels of each transcript were quantified by real-time PCR using SYBR Green detection. The detection of a single product was verified by dissociation curve analysis and relative quantities of mRNA calculated using the method described by [38]. *GAPDH*, *HPRT1* and *C13orf8* relative quantities were used to normalise mRNA levels. For list of primers used, see Table 1.

**Table 1:** Primer sequences use for qRT-PCR.

	Sense	Antisense
ADAM19	GGAAGGACATGAATGGGAAA	CTCGGAACTCTGACACTGGA
C13orf8	AGCAGTGACCAAGAGCAGGT	TCATAGCACGACAGCAACAA
GAPDH	GGTGATGCTGGTGCTGAGTA	TCATAAGTCCCTCCACGATG
HES1	GGTGCTGATAACAGCGGAAT	TTCCAGGACCAAGGAGAGAG
HPRT1	TGGACAGGACCGAACGGCT	TAATCCAACAGGTCGGCAAAG
IGFBP3	CGCTTCGTCTGCACTTGTAA	GGGCGTTCTTCTTCTTGATG
LMO7	TCCTACACGATGGATGATGC	CGAACTGCTCTGGGTTCTCT
SPINT2	CCGGGGCAATAAAAACAACT	CAGGGCAGGATACAACTGCT
TGF-b1	GCCATACTGGCCCTTTACAA	CACGTGCTGCTCCACTTTTA
TGF-b2	CTGGATGCAGCCTATTGCTT	TGGTGAGCGGCTCTAAATCT

### Microarray analyses

Host gene expression was investigated using a custom-designed microarray (Roche NimbleGen Inc., Madison, WI), which represented every bovine RNA reference sequence currently deposited in the NCBI database (n = 26,751). Each gene was represented by two identical sets of five 60-mer-oligonucleotide probes that were isothermal with respect to melting temperature. cDNA was generated from 10 ug total RNA using oligo(dT) primer and tagged with 3′-Cy3 dye and labelled cDNA was hybridised to the array. Gene expression values were calculated from an RMA-normalised dataset [39] and differentially expressed genes were identified using Rank Product Analysis [40]. Genes were defined as differentially expressed using the criteria of a false discovery rate of less than 5% and a fold change of greater than two. Selected gene sets were subjected to hierarchical clustering based on Euclidean distance between expression values and the results were illustrated using a heat-map (ArrayStar3, DNASTAR Inc., Madison, WI).

### Invasion assays of *Theileria*-transformed cells (B2, S3, H7 and Ode cells)

The invasive capacity of the bovine leukocytes was assessed using *in vitro* Matrigel migration chambers, as described [5]. After 26h of incubation at 37°C, filters were washed twice in PBS and the Matrigel was eliminated. In some cases, during this 12h period cells were also incubated with the TGF-b inhibitor SB431542 (10uM), or with recombinant TGF-b protein (10ng/ml). When added TGF-b was maintained in the top chamber, meaning that overall cells were incubated with TGF-b for 36h. Cells were then counted under the microscope (40× objective) to obtain a statistically significant mean number of cells per field (at least 10 fields per filter). The experiment was performed at least in triplicate.

### Time-lapse imaging

Time-lapse imaging using video microscopy was performed with cells growing on glass bottom culture dishes (Willco Wells, the Netherlands) using a Nikon Eclipse TE2000-U inverted microscope equipped with a climate-controlled chamber. Data acquisition and image processing was performed using NIS software of Nikon Instruments. DIC images were acquired in intervals ranging from 0.5 msec to 1 min for 30 min and assembled in movies; acceleration of movies is approximately 600-fold. Kymographs were acquired along a one pixel wide line using NIS software. A 40× Plan Achromat Objective of Nikon was used for longworking distance image acquisition in Matrigel.

### Lamellipodia morphology

*Theileria*-infected macrophages were seeded onto glass coverslips, or glass bottom culture dishes (Willco Wells, the Netherlands) and maintained in growth medium for 48h without or with 10uM SB431542 (SIGMA). SB431542 was replenished after 24h. Cells were either processed for live cell imaging or fixed in 3.5% formaldehyde, 15 min. Actin cytoskeletons of fixed and Triton X-100 permeabilized cells were visualized with Texas red-labelled phalloidin. Lamellipodia area and integrated fluorescence intensities in lamellipodia were determined using photoshop CS3 software.

### Cell seeding onto gelatin/fibronectin matrix

Glass coverslips were coated with 0.1% poly-L-lysine for 15 min at room temperature and then fixed with 0.5% glutharaldehyde for 15min. After 3 washes with PBS, coverslips were inverted onto droplet containing 2mg/ml (0.2%) gelatin (MERCK) in H_2_O for 15min. After 3 washes with PBS, coverslips were inverted onto droplet containing 25ug/ml bovine plasma fibronectin (SIGMA) in PBS and then incubated 1h at room temperature. Coverslips were then transferred into 24 well plate washed once with PBS and kept in PBS until seeding of 50,000 cells per well. After 18h cells were fixed in 3.5% formaldehyde, 15min.

### Embedding cells in 3-D matrices

Embedding in collagen: 3×10^5^ cells in 50ul medium were added to a mixture of 68µl sodium bicarbonate (7.5%, SIGMA), 240ul 10× PBS and 2ml PureCol (3mg/ml, Inamed). The resulting gelatin solution with the concentration of 2.4mg gelatin/ml was distributed into live-cell imaging wells or dishes and transferred to 37°C for collagen polymerization.

Embedding in Matrigel: 1×10^5^ cells in medium were mixed on ice with 250ul growth factor reduced Matrigel (BD biosciences) and was distributed into live-cell imaging wells or dishes and transferred to 37°C for polymerization.

## Supporting Information

Figure S1 A TGF-b autocrine loop leads to constitutive activation of Smad-mediated signalling and increased invasiveness of *T. parva*-transformed B2 cells. **A,** *T. parva*-transformed B2 cells were either incubated for 30min with rboTGF-b1 and rboTGF-b2 (5ng/ml), or treated for 24h with SB431542 (10uM). Nuclear extracts were analysed for phospho-Smad2 and Smad2 levels by Western Blot. PARP protein levels were used as a loading control. **B,** The CAGA-luc construct was transiently transfected into B2 cells and its constitutive activation is given as a relative luciferase activity (arbitrarily set at 100) that represents the ratio firefly/renilla luminescence compared to the minimal MLP promoter. Where indicated, transfected cells were treated for 24h with SB431542 (10uM). CAGA-luc was also co-transfected with a plasmid encoding Smad7 and the degree of inhibition compared to that obtain with an empty vector. Renilla luciferase activity, obtained from a co-transfected pRL-TK vector, was used to correct for transfection efficiencies. **C,** *T. parva*-transformed B2 cells were incubated with or without the SB431542 inhibitor and their invasive capacity was assessed using in vitro Matrigel migration chambers (26h). There was a significant difference between control (DMSO) and treated cells (SB431542) (p = 0.0023). Nine experiments were performed and each sample was counted three times. Data represents the mean number of cells per field (10 fields per well, counted with a 40× objective). The y-axis is the number of migrated cells for 10 independent fields.
(0.21 MB TIF)

Figure S2 Relative expression levels of selected TGF-b target genes in H7 and S3 cells. Total RNA was extracted from H7 cells, S3 cells and S3 cells treated for 24h with rboTGF-b2 (5ng/ml). Relative mRNA levels for the indicated genes were determined by real-time RT-PCR.
(0.08 MB TIF)

Figure S3 TGF regulates actin dynamics in lamellipodia. **A,** *T. annulata* transformed macrophages were seeded onto glass bottom culture dishes and maintained in growth medium for 48h without (Movie S1), or with 10uM SB431542 (Movie S2). Still images of time-lapse image sequences are shown. Arrowheads indicate single lamellipodia on control cells that are absent in SB431542-treated cells. Insets are four fold magnifications of areas highlighted with frames. **B,** *T. annulata* transformed macrophages were fixed in PFA after 48h in culture. Actin cytoskeleton was visualized with Texas red-labelled phalloidin. Insets are four fold magnifications of areas highlighted with frames. **C,** Quantification of lamellipodia area and actin density in lamellipodia from two independent experiments (control: n = 240, SB431542 treated: n = 224). The combination of area and integrated pixel density gives the relative fluorescence intensity for a given area ( = relative actin density).
(0.55 MB TIF)

Figure S4 Membrane blebbing and motility in Matrigel requires Rho-kinase activity. **A,** A still image of live-cell video microscopy analysis of H7 cell embedded for 24h in fibrillar-collagen. A membrane bleb of approximately 5um diameter is highlighted (boxed). Kymograph shows extension and retraction phase of a membrane bleb over a period of one minute. The sharp line at left side of peak (right panel) is the result of the expansion phase being shorter than the interval (3 sec) between images. **B,** Still images of live-cell video microscopy analysis of H7 cells embedded in Matrigel for 24h in the absence (upper) and presence of 3uM H-1152 (lower). **C,** Cell treatment as in B with kymographic analysis of bleb dynamics at leading edge. Each peak in control kymograph represents the complete cycle of a membrane bleb as shown in A.
(1.13 MB TIF)

Movie S1 *Theileria*-infected macrophages were seeded on glass coverslips without treatment. Morphology and motility was recorded by live-cell imaging after 48 h. 600× magnification, 150 min recording time, 1 min intervals and 10 frames/second.
(0.41 MB MOV)

Movie S2 Inhibition of TGF-b receptor signalling with SB431542 affects cell morphology. *Theileria*-infected macrophages were seeded on glass coverslips in the presence of 10 uM TGF-b receptor inhibitor SB431542 (SB43). Inhibitor was re-added after 24 h. Morphology and motility was recorded by live-cell imaging 48 h after seeding. 600× magnification, 150 min recording time, 1 min intervals and 10 frames/second.
(0.50 MB MOV)

Movie S3 Blebbing/amoeboid motility of H7 macrophages in fibrillar collagen. H7 cells were embedded in collagen on glass-bottom cell culture dishes. Cell behaviour was recorded by live-cell video microscopy 24 h after embedding. 600× magnification, 15 min recording time, 3 sec intervals and 10 frames/second.
(1.35 MB MOV)

Movie S4 Blebbing/amoeboid motility of H7 macrophages in fibrillar collagen is blocked after Rho-kinase inhibition. H7 cells were embedded in collagen on glass-bottom cell culture dishes. Rho-kinase inhibitor H-1152 (10 uM) was added 1 h before imaging and cell behaviour was recorded by live-cell video microscopy 24 h after embedding. 600× magnification, 15 min recording time, 3 sec intervals and 10 frames/second.
(1.77 MB MOV)
