# Supplementary figures and images for: TGF-b2 Induction Regulates Invasiveness of Theileria-Transformed Leukocytes and Disease Susceptibility
Source: PLoS Pathog. 2010 Nov 18;6(11):e1001197. doi: 10.1371/journal.ppat.1001197 (PMC2987823; doi:10.1371/journal.ppat.1001197)

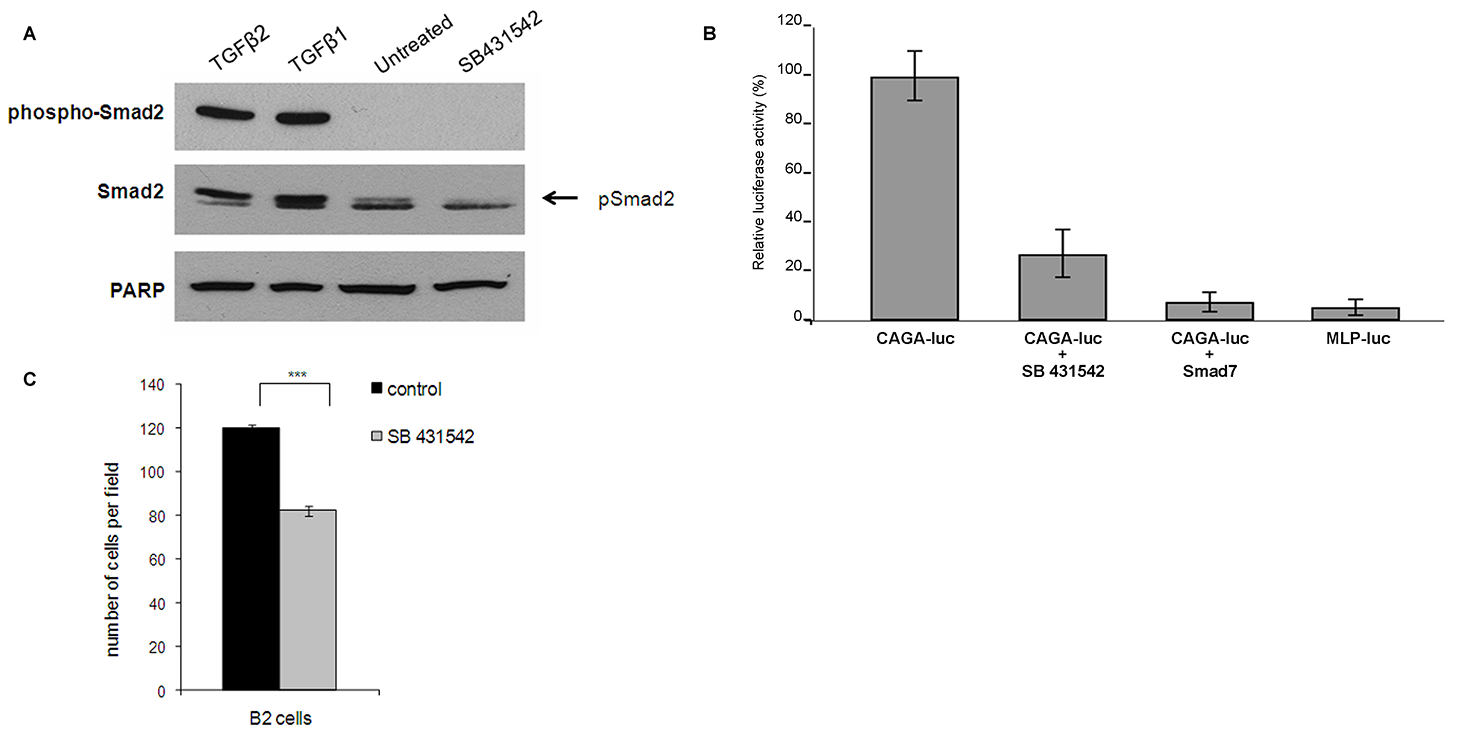

Supplement: Figure S1 — A TGF-b autocrine loop leads to constitutive activation of Smad-mediated signalling and increased invasiveness of T. parva-transformed B2 cells. A, T. parva-transformed B2 cells were either incubated for 30min with rboTGF-b1 and rboTGF-b2 (5ng/ml), or treated for 24h with SB431542 (10uM). Nuclear extracts were analysed for phospho-Smad2 and Smad2 levels by Western Blot. PARP protein levels were used as a loading control. B, The CAGA-luc construct was transiently transfected into B2 cells and its constitutive activation is given as a relative luciferase activity (arbitrarily set at 100) that represents the ratio firefly/renilla luminescence compared to the minimal MLP promoter. Where indicated, transfected cells were treated for 24h with SB431542 (10uM). CAGA-luc was also co-transfected with a plasmid encoding Smad7 and the degree of inhibition compared to that obtain with an empty vector. Renilla luciferase activity, obtained from a co-transfected pRL-TK vector, was used to correct for transfection efficiencies. C, T. parva-transformed B2 cells were incubated with or without the SB431542 inhibitor and their invasive capacity was assessed using in vitro Matrigel migration chambers (26h). There was a significant difference between control (DMSO) and treated cells (SB431542) (p = 0.0023). Nine experiments were performed and each sample was counted three times. Data represents the mean number of cells per field (10 fields per well, counted with a 40× objective). The y-axis is the number of migrated cells for 10 independent fields. (0.21 MB TIF) [file ppat.1001197.s001.tif]

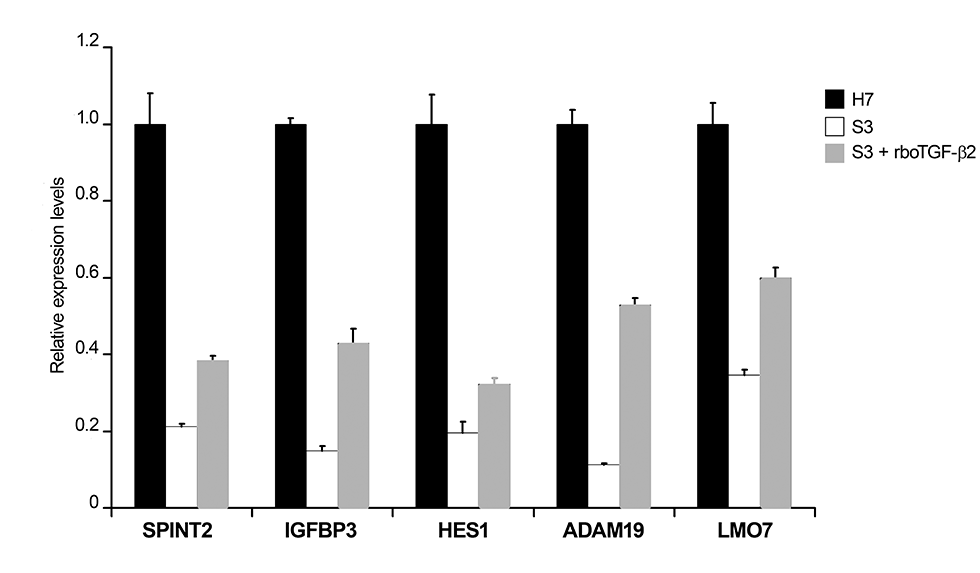

Supplement: Figure S2 — Relative expression levels of selected TGF-b target genes in H7 and S3 cells. Total RNA was extracted from H7 cells, S3 cells and S3 cells treated for 24h with rboTGF-b2 (5ng/ml). Relative mRNA levels for the indicated genes were determined by real-time RT-PCR. (0.08 MB TIF) [file ppat.1001197.s002.tif]

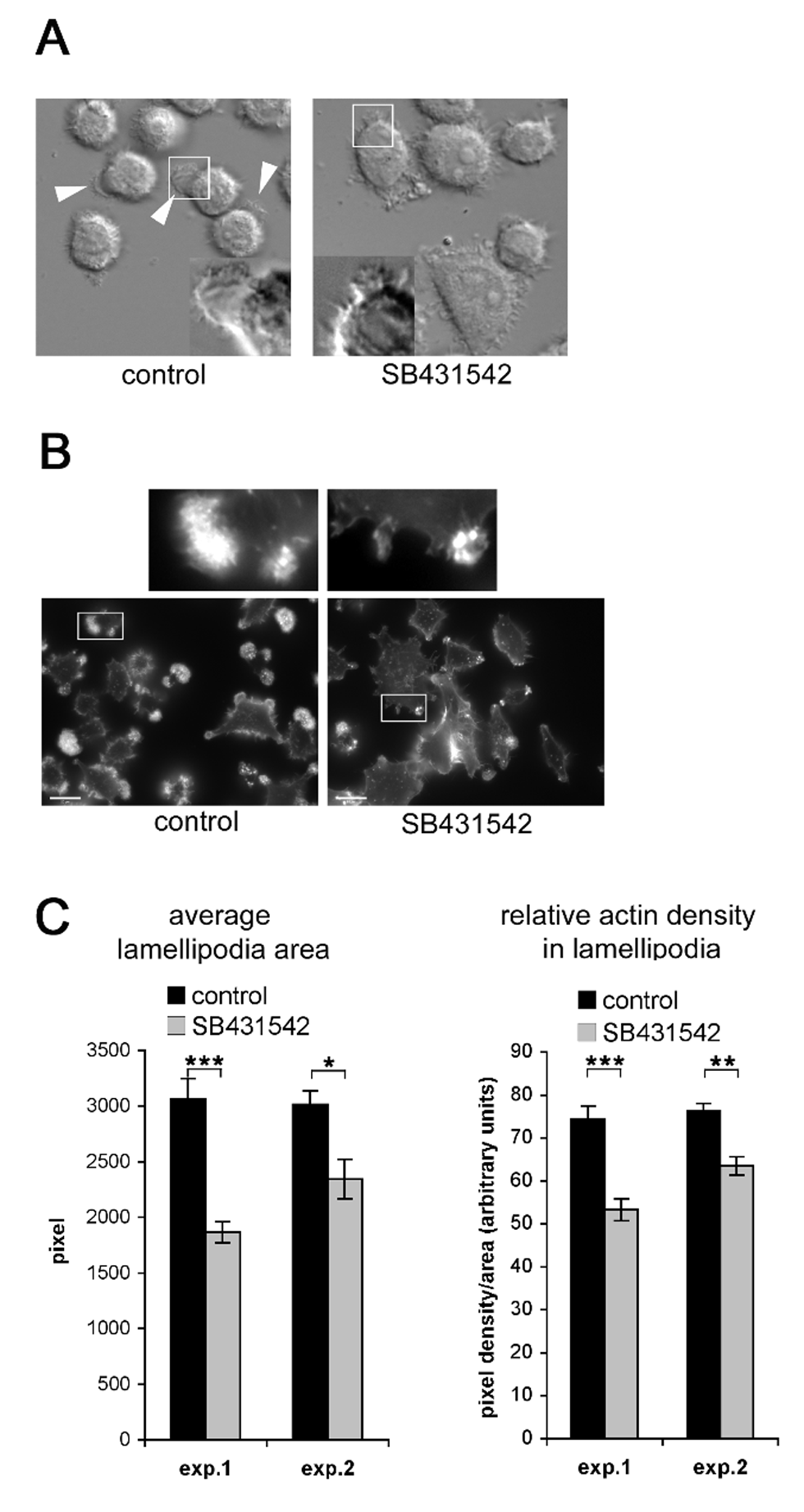

Supplement: Figure S3 — TGF regulates actin dynamics in lamellipodia. A, T. annulata transformed macrophages were seeded onto glass bottom culture dishes and maintained in growth medium for 48h without (Movie S1), or with 10uM SB431542 (Movie S2). Still images of time-lapse image sequences are shown. Arrowheads indicate single lamellipodia on control cells that are absent in SB431542-treated cells. Insets are four fold magnifications of areas highlighted with frames. B, T. annulata transformed macrophages were fixed in PFA after 48h in culture. Actin cytoskeleton was visualized with Texas red-labelled phalloidin. Insets are four fold magnifications of areas highlighted with frames. C, Quantification of lamellipodia area and actin density in lamellipodia from two independent experiments (control: n = 240, SB431542 treated: n = 224). The combination of area and integrated pixel density gives the relative fluorescence intensity for a given area ( = relative actin density). (0.55 MB TIF) [file ppat.1001197.s003.tif]

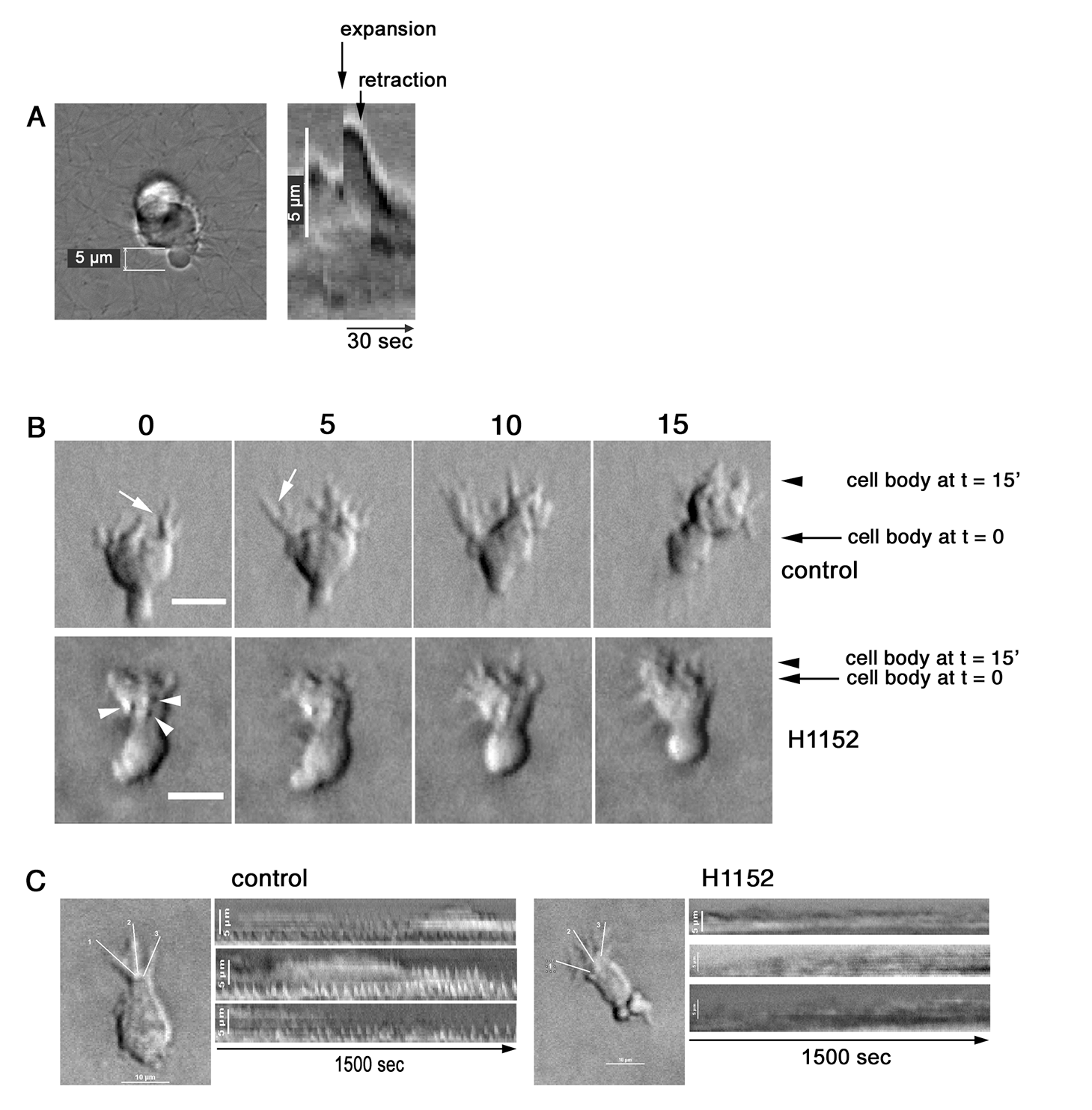

Supplement: Figure S4 — Membrane blebbing and motility in Matrigel requires Rho-kinase activity. A, A still image of live-cell video microscopy analysis of H7 cell embedded for 24h in fibrillar-collagen. A membrane bleb of approximately 5um diameter is highlighted (boxed). Kymograph shows extension and retraction phase of a membrane bleb over a period of one minute. The sharp line at left side of peak (right panel) is the result of the expansion phase being shorter than the interval (3 sec) between images. B, Still images of live-cell video microscopy analysis of H7 cells embedded in Matrigel for 24h in the absence (upper) and presence of 3uM H-1152 (lower). C, Cell treatment as in B with kymographic analysis of bleb dynamics at leading edge. Each peak in control kymograph represents the complete cycle of a membrane bleb as shown in A. (1.13 MB TIF) [file ppat.1001197.s004.tif]
